# Rapid resurgence of syphilis in Japan after the COVID-19 pandemic: A descriptive study

**DOI:** 10.1371/journal.pone.0298288

**Published:** 2024-03-27

**Authors:** Akira Komori, Hirotake Mori, Wenke Xie, Simon Valenti, Toshio Naito

**Affiliations:** 1 Department of General Medicine, Juntendo University Faculty of Medicine, Tokyo, Japan; 2 Department of Emergency and Critical Care Medicine, Tsukuba Memorial Hospital, Tsukuba, Japan; 3 Department of Health Services Research, Faculty of Medicine, University of Tsukuba, Tsukuba, Japan; Universidade Federal do Espirito Santo, BRAZIL

## Abstract

Some countries have reported a post-pandemic resurgence in syphilis prevalence, but trend data in the World Health Organization Western Pacific Region (WHO-WPRO), including Japan, are severely lacking. Thus, the present study compares the number of syphilis cases before and after the COVID-19 pandemic in some WHO-WPRO countries. In addition, temporal trends in the number of syphilis cases in Japan pre- and post-pandemic are described. Annual numbers of syphilis cases during the study periods from China, New Zealand, Australia and Japan were compared. Annual trends of the numbers of syphilis cases during the same study periods were examined in Japan. In 2020, the number of syphilis-positive cases decreased in all four countries. In 2021, though, China, Australia and Japan all showed an increase in the numbers of syphilis cases. However, the rate of increase in China (+2.8%) and Australia (+4.8%) was low compared to Japan (+36.0%). The number of syphilis cases in New Zealand in 2021 was 12.6% lower than in 2020. In 2022, the number of cases of syphilis in China was 7.4% lower than in 2021. The increase of syphilis-positive cases was approximately 6.3-fold higher in Japan compared to Australia (+66.2% vs. +10.5%) in 2022. In conclusion, post-pandemic resurgence of syphilis occurred in Australia and Japan, but not in China and New Zealand. The reason for the substantial increase in syphilis-positive cases in Japan remains unclear. Post-pandemic, prevention and control of sexually transmitted infections still require attention.

## Background

Syphilis is one of the most important sexually transmitted infections (STIs) in the world. The coronavirus disease 2019 (COVID-19) pandemic decreased the prevalence of STIs, including syphilis, worldwide [1, 2]. Some countries, such as the United States and Canada, have reported a resurgence in syphilis after the COVID-19 pandemic [3, 4]. However, detailed data on trends in the prevalence of syphilis in the World Health Organization Western Pacific Region (WHO-WPRO), including Japan, during COVID-19 are scarce. The aims of this study were: 1) to compare the number of cases of syphilis before and after the COVID-19 pandemic in some countries belonging to WHO-WPRO, including China, Australia, and New Zealand, with Japan; and 2) to describe temporal trends in the number of cases of syphilis in Japan before and after the COVID-19 pandemic.

## Methods

### Design and data sources

This retrospective descriptive study used comprehensive surveillance data from the National Institute of Infectious Diseases in Japan (NIID) from January 1, 2018, to December 31, 2022. The NIID is a research institute attached to the Ministry of Health, Labour, and Welfare for conducting (i) fundamental and applied research on infectious diseases and (ii) national testing for lot release and development of antibiotics and vaccines [5]. The NIID provides data about the number of cases of infectious diseases in Japan each year. Permission to use the information made available on the NIID homepage was provided. Regarding other countries, data were obtained for China from the Centers for Disease Control and Prevention of the National Health Commission of the People’s Republic of China [6]. Data from New Zealand were obtained from the Sexually Transmitted Infection surveillance report of the Institute of Environmental Science and Research [7]. Data from Australia were obtained from the National Notifiable Diseases Surveillance System data visualization tool [8]. Information on each country’s website is generally available to the public. There were no changes in case definition criteria of syphilis during the study period.

### Analysis

We combined all cases of syphilis reported by each health agency in each country. The annual numbers of cases of syphilis during the study periods in China, Australia, and New Zealand were compared with those in Japan; all of these countries are members of WHO-WPRO. Then, the annual trends of the numbers of cases of syphilis during the same study periods were examined in Japan. To assess differences by characteristics, the number of cases of syphilis was stratified by age, sex, and sexual preference [men who have sex with men (MSM) or heterosexuals, including both men who have sex with women and women who have sex with men]. We also examined the number of cases of syphilis by classification in Japan. Percentage changes of syphilis-positive cases were calculated as the difference between the number of syphilis-positive cases in the year and that in the previous year divided by the number of syphilis-positive cases in the previous year.

### Ethic approval

Present study is not subject to ethics application. Information that has already been anonymized, has established academic value, is widely used for research, and is generally available to the public.

## Results

**Fig 1:**
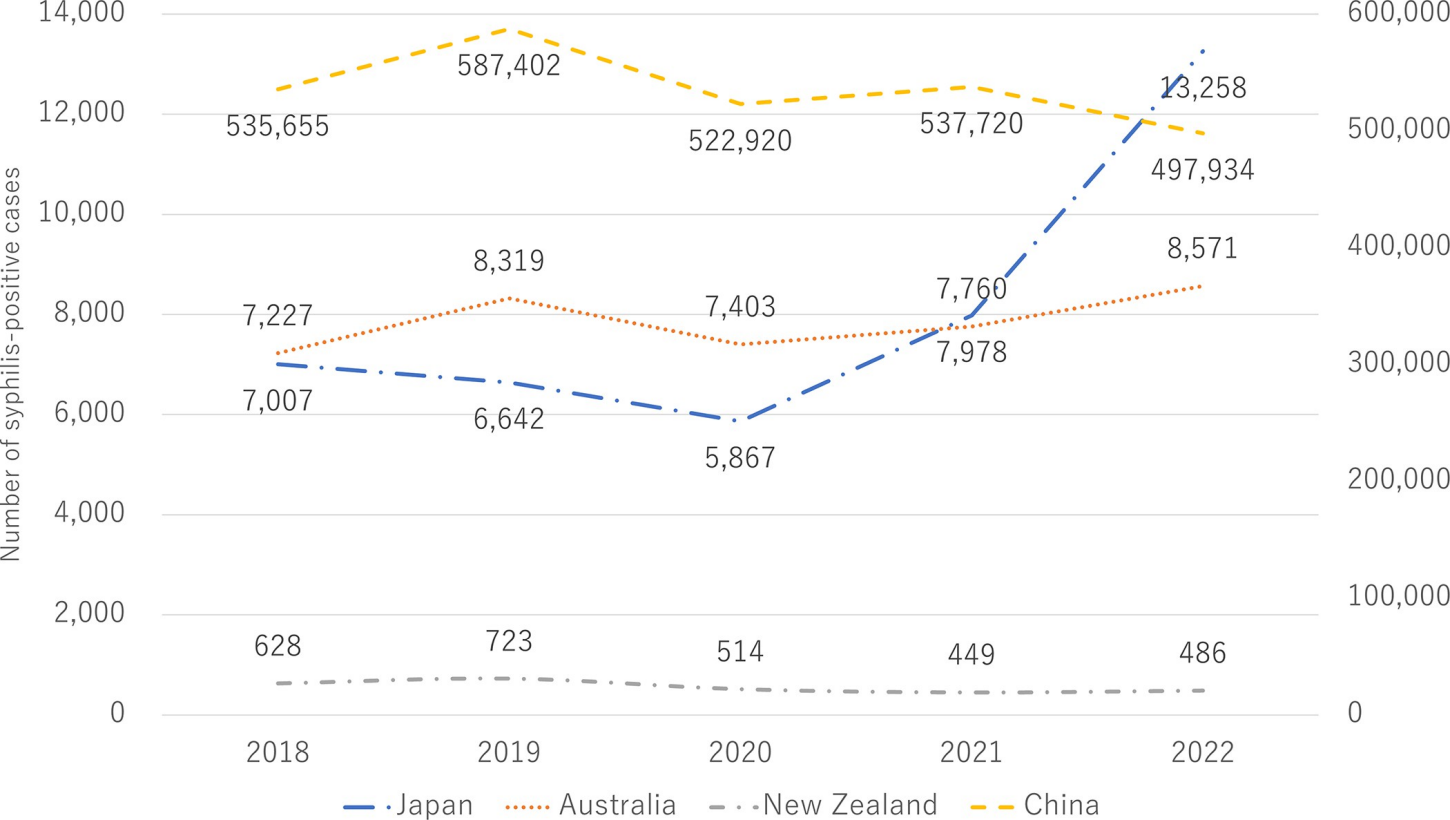
Trends in the numbers of syphilis cases in WHO-WPRO: China, New Zealand, Australia, and Japan. The number beside the line graph indicates the actual number of syphilis-positive cases. The vertical line shows the number of syphilis-positive cases, and the horizontal line shows the time of year.

The trends of the numbers of syphilis cases in China, New Zealand, Australia, and Japan between 2018 and 2022 are shown in Fig 1. In China, New Zealand, and Australia, as in Japan, the number of syphilis-positive cases declined in 2020 when the COVID-19 pandemic occurred: -11.0% in China, -28.9% in New Zealand, -11.0% in Australia, and -11.7% in Japan. One year after the pandemic in 2021, the numbers of cases of syphilis increased in China, Australia, and Japan. Surprisingly, they increased much more in Japan (+36.0%) than in China (+2.8%) and Australia (+4.8%) compared with 2020. In New Zealand, the number of cases of syphilis in 2021 was 12.6% lower compared with 2020. In 2022, the rate of increase of syphilis-positive cases was approximately 6.3 times higher in Japan than in Australia (+66.2% vs. +10.5%). In China, the number of cases of syphilis in 2022 was 7.4% lower than in 2021.

The number of cases of syphilis in each year in Japan is shown in Table 1. The number of syphilis cases in Japan decreased once the COVID-19 pandemic in 2020 occurred, but the number of syphilis cases resurged in 2021 and reached the highest record of 13,258 in 2022 since records began in Japan in 1999. Cases of syphilis were more prevalent in men than in women, and they have increased or decreased similarly for both men and women before and after the pandemic.

**Table 1:** Number of cases of syphilis in Japan: 2018–2022.

		2018	2019	2020	2021	2022
**Total**		7,007	6,642	5,867	7,978	13,258
	Percentage change from the previous year	NA	-5.2%	-11.7%	+36.0%	+66.2%
**Men**		4,593	4,388	3,899	5,260	8,710
	Percentage change from the previous year	NA	-4.5%	-11.1%	+34.9%	+65.6%
**Women**		2,414	2,254	1,968	2,718	4,548
	Percentage change from the previous year	NA	-6.6%	-12.7%	+38.1%	+67.3%

**Fig 2:**
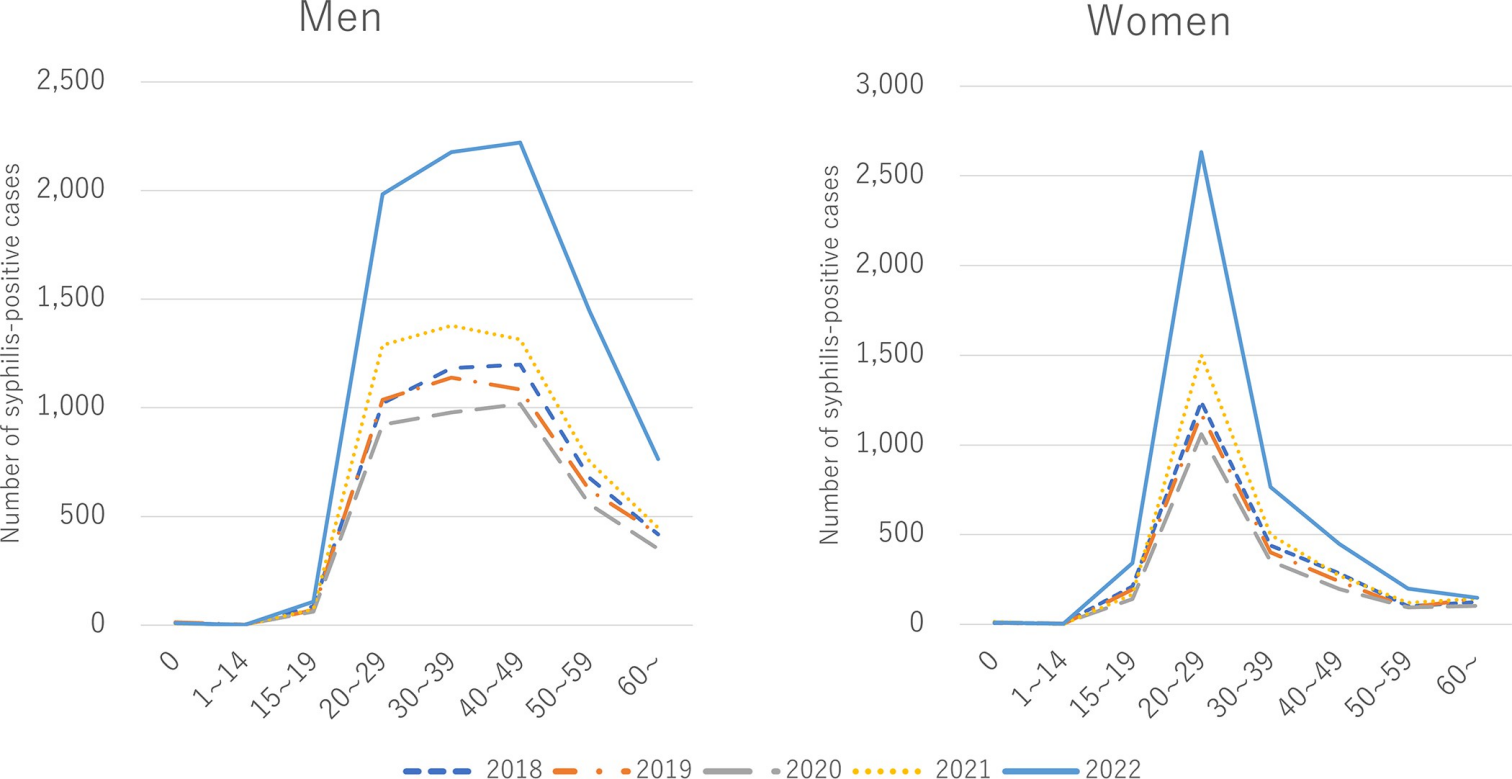
Number of cases of syphilis by age and sex in Japan between 2018–2022. The vertical line shows the number of syphilis-positive cases, and the horizontal line shows age.

Fig 2 shows the number of cases of syphilis for each year stratified by age and sex. Syphilis is most frequently reported in the men in their 20s to 40s and women in their 20s. The trends in the number of syphilis-positive cases by age and sex did not change before or after the pandemic.

**Fig 3:**
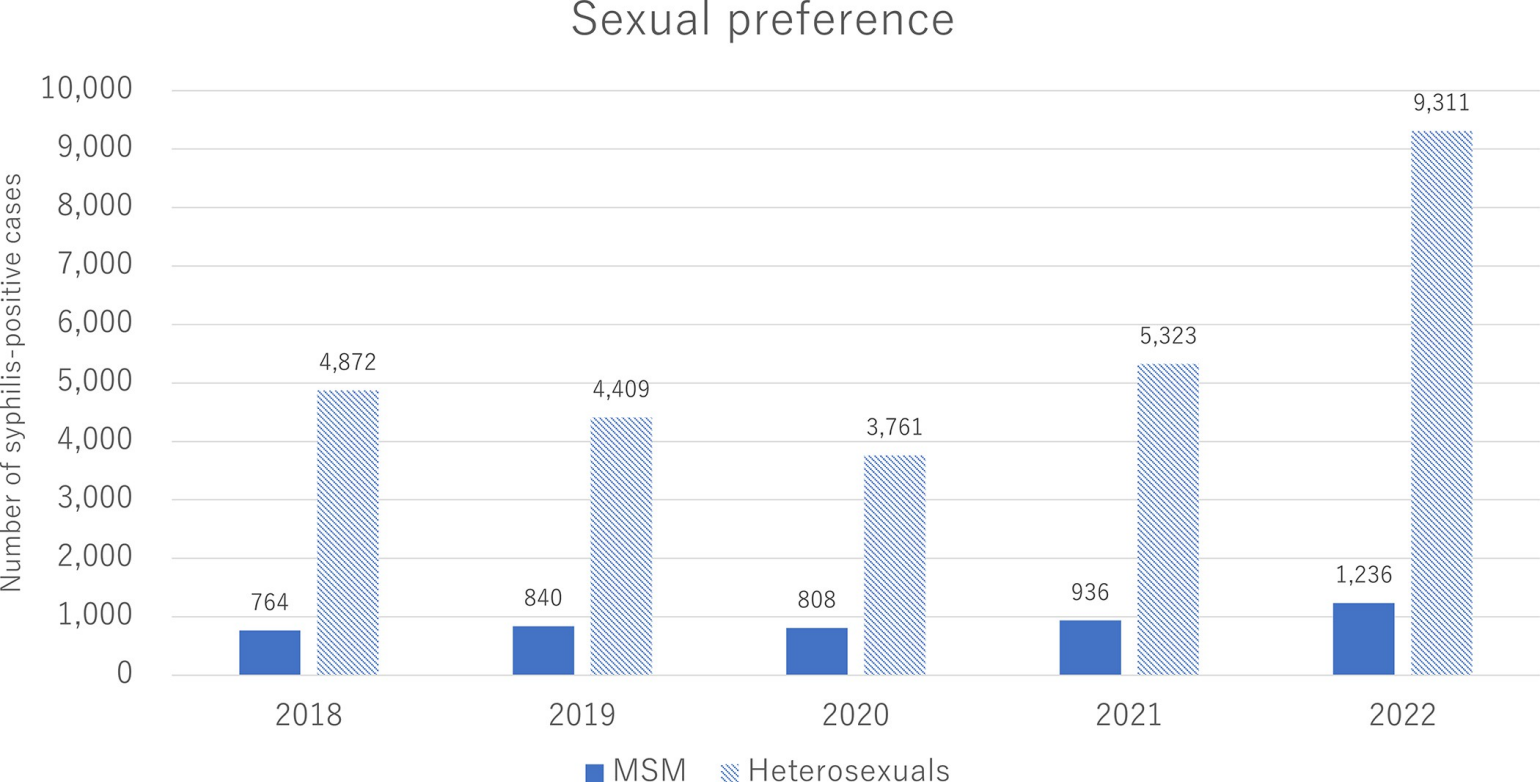
Number of cases of syphilis by sexual preference in Japan: 2018–2022. Heterosexuals include both men who have sex with women and women who have sex with men. The number beside the bar graph indicates the actual number of syphilis-positive cases.

Stratified by sexual preference, the number of syphilis cases increased both in MSM and heterosexuals after the pandemic (Fig 3). However, the number increased more rapidly in heterosexuals than in MSM. The percentage change in syphilis-positive cases from 2020 to 2021 was +15.8% in MSM and +41.5% in heterosexuals, whereas that from 2021 to 2022 was +32.1% in MSM and +74.9% in heterosexuals.

S1 Fig. shows the trends in the number of syphilis-positive cases by syphilis classification in Japan. The proportion of primary and secondary syphilis increased after the pandemic, whereas that of asymptomatic syphilis decreased.

## Discussion

This study examined the change in the number of cases of syphilis in Japan before and after the COVID-19 pandemic and compared the number among WHO-WPRO countries, including China, Australia, and New Zealand, with Japan. The changes in the total numbers of syphilis cases differed among the four WHO-WPRO countries. Resurgence after the COVID-19 pandemic was observed in Australia and Japan, but not in China and New Zealand. Remarkably, the rate of increase of syphilis-positive cases was much more prominent in Japan than in other countries. The reason is not clear. It is unlikely to have been brought in from abroad, since the Japanese government strengthened border restrictions from March 2020 to October 2022, resulting in an over 85% decrease in the number of travellers from abroad compared to before the pandemic [9]. A possible explanation is that the negative impact of stay-at-home behaviour may have been significant in Japan. Syphilis-positive cases in Japan were increasing even before the pandemic [10]. Stay-at-home behaviour might lead to people refraining from visiting clinics for screening [11]. Less screening delayed the detection of syphilis cases and fewer doctor visits led to failure to receive adequate treatment, resulting in spread of infection [11, 12]. Indeed, due to the pandemic, some health centres in Tokyo have suspended testing for diseases other than COVID-19 [13]. Alternatively, as people became less anxious about infection and more accustomed to coexisting with COVID-19, the opportunities for sexual contact may have increased.

In Japan, the change in the number of syphilis cases before and after the pandemic differed by sexual preference; it was less in MSM than in heterosexuals. Less change in MSM might be a characteristic of Japan. A previous study using another Japanese national database showed that the incidence of syphilis in Japan in people living with HIV, who are predominantly MSM, remained stable during the last several years before the pandemic [10]. Alternatively, the reason for the low incidence of syphilis in MSM might be simply because of the lower total number of the MSM population than the heterosexual population. A similar trend was observed in the United States, where the incidence of syphilis increased among heterosexuals, whereas the incidence of syphilis remained stable among MSM [14]. The present result suggests that STI prevention, testing, and treatment services focused on heterosexuals, who are generally not considered to be at high risk for STIs, may be effective in reducing the number of syphilis-positive cases.

The COVID-19 pandemic did not change the trends in the number of syphilis-positive cases by age and sex in Japan. The predominant ages, men in their 20s to 40s and women in their 20s, may reflect frequent sexual activity in these ages [15]. The ages of the highest rate reported of syphilis in Japan is consistent with that in Europe [16] and the United States [14].

The present study warns of the possibility of even more syphilis cases in the future worldwide. Previous studies have suggested that the low incidence of STIs observed during the COVID-19 pandemic might be due to underdiagnosis and underreporting [11, 12]. The prevention and control of STIs like syphilis still needs more attention from society during and after the pandemic.

The present study has some limitations. First, the number of cases of infection may not represent the actual number of patients. Some patients with syphilis might still be unscreened or undiagnosed, resulting in an underestimation of the exact number of patients. Second, data on syphilis from other WHO-WPRO countries were not available. Therefore, the present study does not reflect overall trends of WHO-WPRO countries.

## Conclusions

Trends in the total number of syphilis cases after the COVID-19 pandemic differed among the four WHO-WPRO countries. The number of syphilis-positive cases in Japan had resurgence, with a temporary decline after the COVID-19 pandemic, and the rate of increase in syphilis-positive cases was much greater in Japan than in other countries. Trends in the number of syphilis-positive cases before and after the COVID-19 pandemic in Japan have not changed by age or sex, but they did change by sexual preference. The decrease in the number of patients due to the COVID-19 pandemic may be transient, and future trends should be carefully observed.

## Supporting information

S1 Fig The number of cases of syphilis by classification in Japan showing as 100% stacked bar chart. Heterosexuals include both men who have sex with women and women who have sex with men. The number in the center of graph indicates the actual number of syphilis-positive cases.
(TIF)
