# Supplementary figures and images for: Rapid resurgence of syphilis in Japan after the COVID-19 pandemic: A descriptive study
Source: PLoS One. 2024 Mar 27;19(3):e0298288. doi: 10.1371/journal.pone.0298288 (PMC10971634; doi:10.1371/journal.pone.0298288)

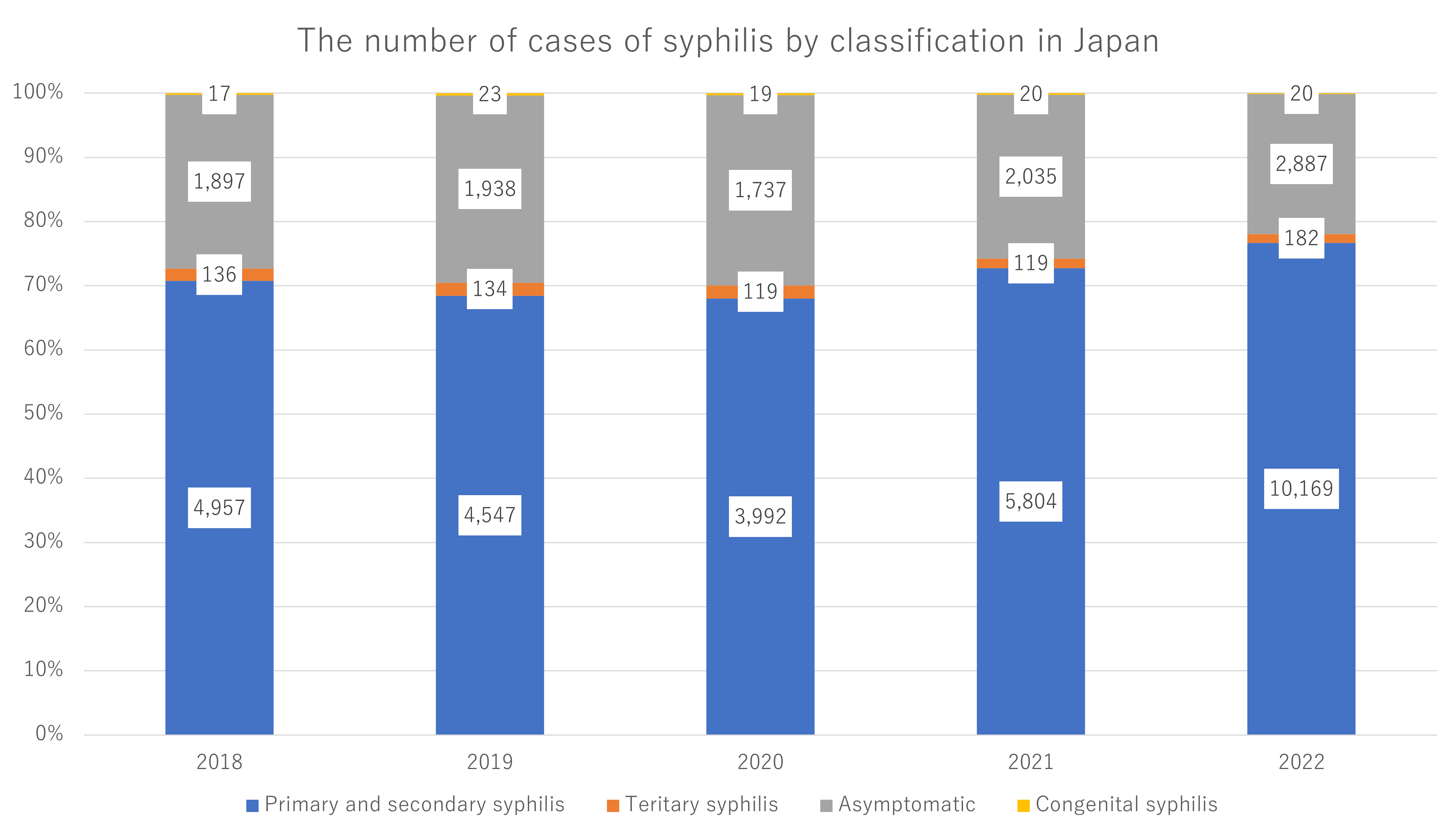

Supplement: S1 Fig — Heterosexuals include both men who have sex with women and women who have sex with men. The number in the center of graph indicates the actual number of syphilis-positive cases. (TIF) [file pone.0298288.s001.tif]
